# *Xenopus* Resources: Transgenic, Inbred and Mutant Animals, Training Opportunities, and Web-Based Support

**DOI:** 10.3389/fphys.2019.00387

**Published:** 2019-04-25

**Authors:** Marko Horb, Marcin Wlizla, Anita Abu-Daya, Sean McNamara, Dominika Gajdasik, Takeshi Igawa, Atsushi Suzuki, Hajime Ogino, Anna Noble, Centre de Ressource Biologique Xenope team in France, Morgane Nicolas, Jacques Robert, Christina James-Zorn, Matthew Guille

**Affiliations:** ^1^National Xenopus Resource, Marine Biological Laboratory, Woods Hole, MA, United States; ^2^European Xenopus Resource Centre, Portsmouth, United Kingdom; ^3^School of Biological Sciences, King Henry Building, Portsmouth, United Kingdom; ^4^Amphibian Research Center, Hiroshima University, Higashihiroshima, Japan; ^5^Centre de Ressources Biologiques Xenopes, CNRS, Inserm, BIOSIT – UMS 3480, Université de Rennes 1, Rennes, France; ^6^Department of Microbiology and Immunology, University of Rochester Medical Center, Rochester, NY, United States; ^7^Xenbase, Division of Developmental Biology, Cincinnati Children’s Research Foundation, Cincinnati, OH, United States

**Keywords:** *Xenopus laevis*, *Xenopus tropicalis*, transgenesis, gene editing, inbred strains, ORFeome, model organism database, resource centers

## Abstract

Two species of the clawed frog family, *Xenopus laevis* and *X. tropicalis*, are widely used as tools to investigate both normal and disease-state biochemistry, genetics, cell biology, and developmental biology. To support both frog specialist and non-specialist scientists needing access to these models for their research, a number of centralized resources exist around the world. These include centers that hold live and frozen stocks of transgenic, inbred and mutant animals and centers that hold molecular resources. This infrastructure is supported by a model organism database. Here, we describe much of this infrastructure and encourage the community to make the best use of it and to guide the resource centers in developing new lines and libraries.

## Introduction

*Xenopus laevis* was first described in 1802 in Daudin’s “Histoire naturelle des rainettes, des grenouilles, et des crapauds” as *Bufo laevis*. For the next century it was used for comparative anatomical studies [reviewed in Gurdon and Hopwood (2000)] but then became widely distributed as a bioassay for human pregnancy (Hogben, 1939). The availability of *Xenopus* underpinned their adoption for the study of biochemical mechanisms driving development and cell physiology (Gurdon and Hopwood, 2000). *Xenopus* has an extraordinary track record as a model organism, playing key roles in discoveries as disparate as isolation of the first eukaryotic gene and transcription factor, the first demonstration of nuclear reprogramming and the discovery of the mesoderm inducing and Spemann organizer molecules, as reviewed in Harland and Grainger (2011) and Tandon et al. (2017). More recently, the size and number of its synchronous embryos, its blastomere size, its well-defined fate map (Shindo et al., 1987) and accurate genome sequences (Hellsten et al., 2010; Session et al., 2016) have enabled the allotetraploid *X. laevis* and *X. tropicalis*, its diploid relative, to be used very successfully for transcriptome (e.g., Briggs et al., 2018), proteome (e.g., Baxi et al., 2018), and metabolome studies. Together with the genetic resources described below, and the high efficiency of gene editing in these species, which allows very rapid studies of human genetic disease variants, these techniques continue to demonstrate the value of *Xenopus* as key model organisms. Here, we review the various resources that are in place to underpin and support research using the *Xenopus* models.

## Transgenic Resources

Transgenesis, or the ability to transfer DNA from one genome to another, is a powerful tool which can be used in established model systems for the study of regulatory and coding DNA in normal and disease-associated processes as well as changes in gene function and control that occur during evolution. The origins of transgenesis can be traced back to the discovery of bacterial restriction enzymes and their use to generate recombinant DNA plasmids in the early 1970s (Cohen et al., 1973). In *Xenopus*, a number of reports in the 1980s demonstrated that simply microinjecting exogenous plasmid DNA into fertilized eggs can result in its successful integration into the frog genome (Rusconi and Schaffner, 1981; Etkin and Roberts, 1983; Andres et al., 1984; Bendig and Williams, 1984; Etkin et al., 1984), although showing mosaic distribution in the adult tissues. Etkin and Pearman (1987) demonstrated that exogenous, linearized plasmid DNA can be successfully transmitted through the germline of the transformed male parent to following generations; however, this approach proved to be highly inefficient since only ∼1% of the injected individuals demonstrated mosaic integration of the DNA into their germline (Yergeau et al., 2010). It took another decade for the idea of using *Xenopus* in transgenic studies to become a practicable endeavor with the development of much more efficient methods of transgenesis, initially via restriction enzyme mediated integration (REMI) (Kroll and Amaya, 1996) and then through the use of phiC31 integrase (Allen and Weeks, 2005), I-SceI meganuclease (Ogino et al., 2006; Pan et al., 2006), and various transposable element-based approaches (Yergeau et al., 2007). The use of I-SceI meganuclease has been shown to be especially effective with the reported ratios of non-mosaic integration in the F0 generation and germline transmission as high as 30% in *X. tropicalis* and 20% in *X. laevis* (Ogino et al., 2006). These transgenesis methods result in random integration of the exogenous DNA and, although approaches for targeted, precise integration using gene editing have recently been described (Aslan et al., 2017), their use in the *Xenopus* community is at an early stage (see below).

Several practical aspects make *Xenopus* an enticing model to use in transgenic studies. These include the ability of a single female to produce as many as 4000 eggs per spawning (Wlizla et al., 2017), thus providing a large batch of sibling embryos that are synchronous and develop externally. Furthermore, embryonic development is relatively rapid, with most major organs formed within 5 days following fertilization, and is easily observable since the tissues surrounding major viscera are transparent during the same time frame (Nieuwkoop and Faber, 1994; Khokha et al., 2002). However, the model is somewhat limited by the generation time with the two most commonly used species, *X. laevis* and *X. tropicalis* taking approximately 6–12 and 5–8 months, respectively, to reach sexual maturity, with males maturing slightly faster than females. Due to this limitation, most individual labs tend not to spend their time and resources to generate true breeding transgenic animal lines and instead focus on experiments that take advantage of the rapid early development. The disadvantage of a long generation time is to some extent balanced by the long period of fertility in the animals with *X. laevis* as old as 15 years producing viable offspring (Tinsley and Kobel, 1996); careful breeding strategies can thus be used to avoid significant genetic drift in this species.

The *Xenopus* resource centers including the National BioResource Project (NBRP) in Japan, the European *Xenopus* Resource Centre (EXRC) in Europe, and the National *Xenopus* Resource (NXR) in the United States of America were established, in part, to serve as centralized repositories with sufficient infrastructure to allow for maintenance of the extant *Xenopus* transgenic lines at capacities allowing for their distribution to individual labs on an as needed basis (Pearl et al., 2012). The stock centers also have expertise in generating new lines which can then be grown and made available to the research community. This has effectively eliminated the need for labs to contribute crucial resources into generation of novel transgenic lines since these are readily available for distribution as adults, tadpoles, embryos, isolated testes, or cryopreserved sperm (Pearl et al., 2017). Currently, the stock centers hold over 130 different transgenic lines, a number that is continuously increasing, and which can be grouped into four different categories: (1) reporter expression lines, (2) inducible lines for disruption and regulation of signaling pathway activity, (3) GAL4 and Cre driver lines, and (4) single landing site lines (Supplementary Table S1).

Reporter expression lines form by far the largest category of transgenic *Xenopus* lines available and can be further subdivided into several groups. First are the tissue/region specific lines which typically contain a fluorescent protein driven by a specific promoter to mark a particular tissue, region, or organ in the developing embryo (Supplementary Table S1A). Besides being useful for observation of normal development, these lines are highly amenable to investigations of abnormal development following disruption of gene activity, as demonstrated by a recent study from the Miller lab using a line marking the developing kidney, *Xla.Tg (Dre.cdh17:eGFP)^NXR^* (**RRID:NXR_0102**), to investigate the disruption in kidney development following morpholino induced knockdown of the Planar Cell Polarity pathway component, Daam1 (Corkins et al., 2018). Studies using a similar design can easily be performed in lines marking other parts of the developing embryo taking advantage of the other tissue/region specific lines available. Certain experimental conditions, in particular ones relying on the use of formalin-fixed, paraffin-embedded tissues, result in a considerable loss of true fluorescent protein signal, and an increase in tissue autofluorescence (Jiang et al., 2005; Swenson et al., 2007). In cases where such histological preparations are necessary, transgenic lines that mark tissues/regions through colorimetric methods can be used in place of fluorescent reporters. An example of such a line is *Xtr.Tg (tubb2b:Has.ALPP;cryga:dsRed)^Amaya^* (**RRID:EXRC_3003**, **NXR_1099**), which allows for rapid detection of axonal projections *in situ* by a simple alkaline phosphatase reaction at any stage of development in whole embryos, which can then be further processed for histology (Huang et al., 2007). This reporter group also includes lines driven by ubiquitous promoters like CMV and human ubiquitin C. These are particularly useful for cut-and-paste, transplantation-based experiments to label and fate map regions of host embryos.

The second group of reporter expression lines includes transgenics marking subcellular organelles (Supplementary Table S1B). These are highly useful for the study of molecular processes involved in cell function and are especially effective when utilized in the context of *Xenopus* egg extracts, the only cell-free system that permits full investigation of all DNA transactions related to cell cycle progression and DNA damage repair (Cross and Powers, 2009; Hoogenboom et al., 2017). Many of these lines have been generated by the Ueno lab and mark a diverse range of organelles including plasma membrane, *Xla.Tg (CMV:RFP-CAAX)^Ueno^* (**RRID:EXRC_0075**, **NXR_0115**), golgi bodies, *Xla.Tg (CMV:Has.B4GALT1-eGFP)^Ueno^* (**RRID:EXRC_0077**, **NXR_0110**), and microtubule plus ends, *Xla.Tg (CMV:Has.MAPRE3-eGFP)^Ueno^* (**RRID:EXRC_0078**, **NXR_0109**) (Takagi et al., 2013). Lines generated by other labs, as well as by the stock centers themselves, that label other organelles are also available.

The third group of reporter lines are those that serve as indicators of signaling activity (Supplementary Table S1C). These include Wnt signaling reporter lines generated by the Vleminckx lab in both *X. laevis* and *X. tropicalis* (Tran et al., 2010), an apoptosis sensor (Kominami et al., 2006), a histone H3 lysine 9 acetylation sensor for *in vivo* epigenetic analysis (Sato et al., 2013; Suzuki et al., 2016), a neural tissue specific calcium signaling sensor (Chen et al., 2013), and two distinct lines for detection of oxidative stress response (Love et al., 2013).

The fourth group of reporter lines are transposable element enhancer trap lines, all generated by the Mead lab (Supplementary Table S1D; Yergeau and Mead, 2009; Yergeau et al., 2010). Although the integration sites for some of these lines have been identified, this is not the case for all of them. These lines are of use in studies where the particular promoter driving expression is not important and it is the labeled region of the embryo that matters. They can also be used for further study of transposable element activity and additional discovery of regulatory regions via enhancer trap approaches and include lines which result in remobilization of the transposons in the offspring produced.

The fifth group currently contains a single line generated in the Buchholz lab allowing for temporal regulation of fluorescent marker expression via treatment with doxycycline (Supplementary Table S1E; Rankin et al., 2009). Other lines where temporal transgene expression can be regulated through use of simple small molecule treatments or heat shock will likely be added to this group in the future.

The final group includes reporter lines that are especially suitable in fate mapping studies via temporal or regional switch in fluorescence (Supplementary Table S1F). These lines work best when used in crosses with transgenic driver lines designed to regulate the switch in expression pattern (Supplementary Tables S1, S3). Among these reporters are two Brainbow lines generated at the NXR as well as a number of lines made in the Ryffel lab (Waldner et al., 2006; Livet et al., 2007). The initial fluorescence is ubiquitous; however, these lines contain loxP or FRT sites, and the initial fluorescence can be altered through crosses with driver lines that express Cre or FLP recombinase, respectively. The change in fluorescence can be spatially or temporally regulated through use of region specific, or inducible promoters or can alternatively be induced by targeted microinjection of *Cre* or *FLP* mRNA. This group of reporters also includes two types of lines which rely on a binary effector-transactivator design to function, the GAL4-UAS system and the tet-on system (Gossen and Bujard, 1992; Chae et al., 2002; Hartley et al., 2002).

In the GAL4-UAS system, the reporter gene is regulated by a UAS effector and will only be expressed when crossed to a driver line expressing GAL4. This allows for spatial or temporal control of reporter expression dependent on the promoter used to control expression of GAL4. Similarly, in the tet-on, the reporter is regulated by a TRE effector and will only be expressed if crossed to a driver line containing an rtTA transactivator. However, besides the promoter used to control the rtTA expression, an additional level of temporal control is provided since rtTA only works in the presence of doxycycline.

The remaining three main categories are somewhat less varied than the reporter lines but have important functional applications and include: inducible lines for disruption and regulation of signaling pathway activity (Supplementary Tables S1, S2), GAL4 and Cre driver lines (Supplementary Tables S1, S3), and single landing site lines (Supplementary Tables S1, S4). Disrupting signaling pathways can be achieved by microinjection of constitutively active or dominant negative pathway components into early embryos, however, many of the transgenic lines available are designed to allow for precise temporal control thus permitting the study of signaling pathway roles at much later stages of development than microinjection allows.

There are two ways these transgenes are regulated. First is through use of a heat shock promoter as in the *Xla.Tg (hsp70:nog;cryga:GFP)^Jmws^* (**RRID:EXRC_0018 NXR_0020**), which was used to study temporal requirement for BMP signaling in haematopoiesis (Kirmizitas et al., 2017), or the *Xla.Tg (hsp70:Xtr.dkk1;cryga:GFP)^Jmws^* (**RRID:NXR_0021**), which could be used in a similar manner to interfere with Wnt signaling. The second way the expression of these transgenes can be regulated is through use of binary control systems like CRE-Lox, FLP-FRT, GAL4-UAS, and tet-on. These lines may require the use of driver transgenic lines to regulate their activity, but not always, as in the case of *Xla.Tg (Mmu.col1a2:rtTA;TRE:DNthra-GFP)^Brown^* (**RRID:EXRC_0026**) which has both components of the tet-on system and allows, in the presence of for expression of a GFP tagged dominant negative form of the thyroid hormone receptor (DNthra) specifically in cartilage, and which was used in the study of thyroid hormone function in limb development (Brown et al., 2005).

The currently available driver lines allow for use of CRE or GAL4 in a manner described above in order to control changes in fluorescence patterns of reporter lines or activities of signaling. In *X. laevis* the CRE drivers are mainly designed to allow for temporal control through the use of a heat shock promoter or doxycycline treatments as part of a tet-on system, although a transgenic line from the Ryffel lab that allows for CRE expression specifically in muscle cells is also available (Waldner et al., 2006; Roose et al., 2009; Kerney et al., 2012). In *X. tropicalis* there is a GAL4 driver line which allows for both temporal and spatial control, *Xtr.Tg (tubb2b:PR-Gal4;cryga:CFP)^Zimml^* (**RRID:EXRC_3033**, **NXR_1109**). The *tubb2b* promoter, used here, drives expression specifically in neural cells, however, the GAL4 DNA binding domain is fused to the ligand binding domain of the progesterone receptor and thus, to function, requires the progesterone antagonist RU-482. This allows for full temporal regulation (Waldner et al., 2006). When used together with some of the available UAS effector lines temporal disruption of Wnt or Hedgehog signaling can be induced specifically in the nervous system. Additional driver lines that allow expression in other tissues and the use of FLP-FRT or the tet-on systems will very likely become available in the future.

Finally, there is the landing site category which currently contains a single line, *Xla.Tg (CFP-ATTP)^Ryff^* (**RRID:EXRC_0058**). This line contains an *attP* docking site within a functional blue fluorescent protein coding sequence. Using phiC31 mediated integration, exogenous proteins can be introduced into this line, and loss of blue fluorescence can be used to screen for effective integration. Transgenic animals generated this way are likely to show very similar levels of the exogenous proteins expressed, due to local transcriptional control elements and chromatin environment being the same.

The transgenic lines available from the stock centers can be used in versatile ways to investigate many questions that are essentially low hanging fruit, ready to be plucked. The whole list may be a bit overwhelming at first glance, but the staff at the centers is there to provide advice and guidance regarding the best lines available for use in the study of particular questions. Individual investigators are encouraged to take advantage of this expertise and the lines available to move their research forward. Furthermore, in particular the EXRC and the NXR have expertise in generating novel transgenic lines and growing them to adulthood. If a line of interest is not available, we encourage investigators to take the initiative and request it to be made or provide guidance to aid the stock centers in determining which lines should be generated as a priority.

## Gene Editing Resources

*Xenopus* have proven to be excellent organisms in which to perform gene editing experiments due to their external fertilization and efficiency of RNA and protein microinjection into synchronous embryos to deliver ZFNs (Nakajima et al., 2012; Young and Harland, 2012), TALENS (Lei et al., 2013; Miyamoto et al., 2015), or CRISPR/Cas9 (Blitz et al., 2013; Nakayama et al., 2013). Numerous labs have shown that production of simple insertions and deletions (indels) is very straightforward, fast, and efficient (McQueen and Pownall, 2017; Tandon et al., 2017). Studies using CRISPR/Cas9 also allow loss of function experiments to take place much later than those previously possible in *Xenopus*, which relied on injection of mRNAs expressing dominant negative proteins (Amaya et al., 1991) or antisense morpholino oligonucleotides that have recently been shown to be able to cause off-target, generic, phenotypic effects (Gentsch et al., 2018). The main drawback to gene editing in *Xenopus* is that, in F0 animals, it produces mosaicism (Ratzan et al., 2017); this is mainly due to the rapid cell divisions that occur every 30 min and the low temperature at which *Xenopus* are raised. Frogs made this way have nonetheless been used to address various biomedical topics, including cancer, immunology, neurobiology, cell biology, and other developmental biology questions (Naert et al., 2016; Banach et al., 2017; Hassnain Waqas et al., 2017; Delay et al., 2018) as well as to provide embryos that can be used as tools to understand human genetic diseases (Feehan et al., 2017; Deniz et al., 2018; Naert and Vleminckx, 2018a; Sega et al., 2018). Although mosaic, mutations in F0 animals are useful due to the high level of penetrance, often exceeding 90% in *X. tropicalis* when measured using TIDE (Brinkman et al., 2018; Naert and Vleminckx, 2018b).

**FIGURE 1:**
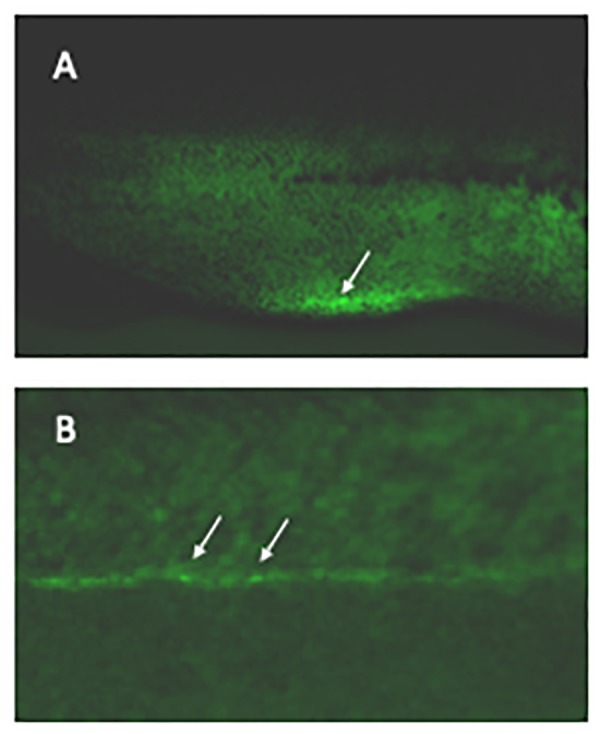
Labeling haematopoietic stem cells in live embryos. *Xenopus laevis* eggs were microinjected with CRISPR/Cas9 targeting the 3′UTR of *runx1* together with a DNA construct containing 400 bp homology arms from either side of the cut site flanking an IRES controlling eGFP expression. Founder embryos were screened for mosaic expression of GFP in the correct regions and 24 grown to adulthood. Their offspring were then screened for germline transmission; two sets of offspring showed strong expression in the vbi (**A**, arrowed) and vasculature (**B**, examples arrowed) as expected for *runx1* at this stage. The transmission rate was 46 and 52% in the 2 sets. An F2 embryo is shown.

In addition to indel-based knockout F0 animals, the resource centers are also producing knockout lines both for the community and to order. Staff in the centers work closely with individual researchers to create mutants in specific target genes and then breed the animals to determine germline transmission. In addition, a large program targeting 200 developmentally important genes prioritized by the research community is being undertaken at the NXR, although it is funded separately from the resource center itself. The first of these lines are now being inbred to produce F2 homozygous mutant animals and knockout phenotypes are being assayed; phenotypic analysis is then done in collaboration with individual researchers. These mutants are being made available and cataloged on Xenbase as soon as they are initially characterized. Making locus-specific gene knock-ins in embryos has, in all vertebrates, proven a significant challenge. Although this is possible in *Xenopus* by using injection of fertilized eggs and screening large numbers of animals (for example in the *runx1* locus shown in Figure 1), it is inefficient and there are no reports of precise DNA construct integration in the germline by egg injection. Recent work shows, however, that being able to access the *Xenopus* oocyte to apply gene editing techniques for insertion largely overcomes this challenge (Aslan et al., 2017), most likely because the levels of the homology directed repair (HDR) machinery are much higher in oocytes than eggs (Hagmann et al., 1996). To prevent mosaicism, oocytes can be cultured long enough (3 days) for injected sgRNA and Cas9 to decay before fertilization and treatment with SCR-7, a DNA ligase IV inhibitor, and increases the likelihood that the genetic lesion is repaired via HDR mechanisms instead of the double-strand break repair pathway (DSBR) (Aslan et al., 2017). There are other approaches being taken in several organisms to improve HDR-mediated integration using CRISPR/Cas9 and it should become clear in the near future which becomes the dominant technology in *Xenopus*.

## Robust Inbred Strains of *X. tropicalis*

There are two major wild type strains of *X. tropicalis*: “Ivory Coast” and “Nigerian” that were originally collected from different localities (Tymowska and Fischberg, 1973; Grainger, 2012). After their introduction to the community in 1990 they were bred in several different labs, resulting in a number of strains that can be distinguished by mitochondrial haplotype and genotypic data on SSLP markers. The Ivory Coast and Nigerian strains are evolutionally diverged, and some Ivory Coast strains have diverged from one another while Nigerian strains share a unique mitochondrial haplotype (Kashiwagi et al., 2010; Igawa et al., 2015). Some of these strains are particularly suited to genetic studies, for example those with a short generation times such as Golden and Superman.

**FIGURE 2:**
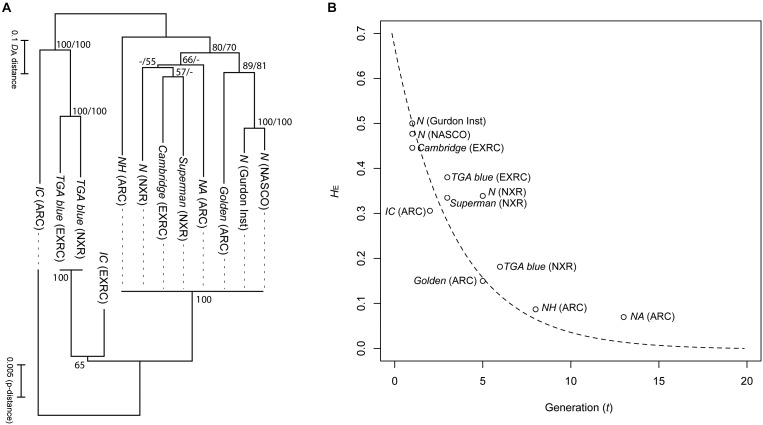
Genetic relationships and heterozygosity reduction curve in the strains of *X. tropicalis*. **(A)** Neighbor joining trees of the strains based on genetic distances (*D*_A_) of 60 Simple Sequence Length Polymorphism (SSLP) markers (upper) and p-distance of mitochondrial haplotypes (2,328 bp; lower). Numbers on branches indicate percent bootstrap probability (only indicated > 50%). **(B)** Plot of expected heterozygosity (*H*_E_) against generation number (*t*). The curved dotted strain indicates theoretical reduction of heterozygosity via single brother-sister matings for every generation, calculated from the following equation: *H_E_* = *H*_0_${\left(1-\frac{1}{2N_{e}}\right)}^{t}$.

When using reverse genetic tools such as CRISPR/Cas9, that rely on a lack of polymorphisms in target regions for cutting, sequence insertion and analysis, inbred strains greatly increase the efficiency of experiments. Four strains in the NBRP *X. tropicalis* have been successfully maintained to achieve inbred status (Igawa et al., 2015). Heterozygosity value (*H*_E_) of SSLP markers decreased following a theoretical reduction curve due to brother-sister mating in every generation (Figure 2). The offspring are vigorous in viability, comparable to other non-inbred strains, and thus these strains have stabilized past the stage of inbreeding depression. The inbred strains have been tested successfully for transgenesis and gene editing (Sakane et al., 2018) and should begin to become available to the community late in 2019.

## Training Resources

As described above, new genetic and biochemical techniques are being developed for *Xenopus* research, and the application and usability of *Xenopus* in basic and biomedical sciences are expanding into a wide range of research areas such as human disease modeling in CRISPR/Cas9 induced mutants. To meet this increasing demand, the resource centers are not only distributing materials but also providing a variety of advanced training courses and research opportunities for users who are not familiar with basic or advanced methods. Each resource center organizes a unique combination of educational opportunities and updates the content of courses annually based on requests from users.

In 2013, the NBRP *X. tropicalis* started running annual technical courses in Hiroshima, to teach several basic techniques such as artificial fertilization of eggs, microinjection of synthetic mRNAs/oligonucleotides, and husbandry of tadpoles and frogs. Trainees include faculty members, post-doctoral researchers, and graduate and undergraduate students from across Japan. The training course has been continually updated, and now includes transgenesis, genome editing and bioinformatics. Moreover, a user survey resulted in further development of the course in 2018. To allow more time for genome editing techniques, the NBRP technical courses are now held in both summer and winter annually. The technical courses represent a good opportunity not only for providing training, but also for distributing and testing genetic tools – more than 200 plasmid samples from the *X. tropicalis* cDNA plasmid collection and transgenesis plasmids have been distributed during the past six courses. In addition to its technical courses, the NBRP *X. tropicalis* has been organizing a joint technical course with the NBRP Medaka in Okazaki, Japan. This joint course is designed for overseas trainees from around the world and aims to facilitate the beneficial use of two different aquatic model organisms. *Xenopus* has become a popular animal for education in schools, members of the NBRP *X. tropicalis* have been providing opportunities to introduce *Xenopus* research to medical university and high-school students across Japan and to students from other Asian countries. To reach a wider range of researchers, the NBRP *X. tropicalis* is also running XenoBiores^1^, which facilitates the exchange of questions and troubleshooting among users and provides the latest information on a variety of topics including conferences reports, technical courses and genetic/live-animal resources.

The NXR at the Marine Biological Laboratory (MBL) in Woods Hole, has a range of advanced training courses, which are run annually and are aimed at advanced and beginner *Xenopus* researchers. These courses bring experts in the fields of bioinformatics, specialized imaging, and gene editing to teach cutting edge techniques to the participants. The Bioinformatics Workshop is designed for wet lab biologists who want to extend their understanding of computational tools and methods and may also want to acquire grounded computational skills to enable them to work independently. The imaging workshop provides hands on training in imaging *Xenopus* embryos, including live imaging of embryos and explants and image processing, analysis and quantitation. The genome editing workshop focuses on providing theoretical and practical aspects of genome editing in *Xenopus*, allowing participants to generate mutants during the workshop. Husbandry training is also offered at the NXR to promote harmonization among *Xenopus* researchers and improve yields of healthy and productive frogs throughout the community. Furthermore, the NXR offers a research facility service, which allows visiting scientists to integrate themselves in the NXR facility and MBL. This allows full access to all available *Xenopus* lines, equipment, reagents, and expertise in gene editing. Uniquely, the NXR allows for a rich collaborative environment established by the diverse group of scientists from different disciplines who come every year to do their research at the MBL.

The EXRC at Portsmouth does not run specific training courses, but within its remit is a “research hotel” service that is becoming heavily used. Researchers, who may be very familiar with *Xenopus* or used to other models, come to the center and work alongside staff to learn new techniques and carry out their own experiments, using all of the resources at cost. Often, they make new lines to be grown up in the center then return to analyze them.

## Specialized *Xenopus* Resources

Also situated in Europe and regularly providing training is the French *Xenopus* Biological Resource Center (CRB^2^), which has been located in Rennes since 2008. Training is provided by members of the CRB in the fields of *Xenopus* breeding techniques, embryo manipulation and, like at the EXRC, how to care for *Xenopus* as required by EU laws. Research-wise, the CRB has special expertise in projects centered on the screening of biologically active compounds in *Xenopus* oocytes with electrophysiological assays; the center is equipped to perform this type of analysis at high throughput using robotic approaches. The CRB can also offer transgenesis, CRISPR/Cas9 for targeted knockouts and screening of the mutations. Many of the wild-type *X. laevis* and *X. tropicalis* used in Europe are provided by the CRB and it is closely involved with projects to improve the health of aquatic laboratory animals. Shortly the CRB will relocate to the biomedical campus in Rennes. Excitingly, this relocation should involve the development of a state-of-the-art facility for the development and housing of transgenic animals.

The *X. laevis* research resource for immunobiology (XLRRI^3^) at the University of Rochester Medical Center, is a comprehensive research resource specializing in the use of *X. laevis* as a multi-faceted experimental platform for immunological research. The XLRRI maintains and provides to the research community MHC-defined inbred strains and clones of frogs, as well as tools such as lymphoid tumor cell lines, MHC-defined fibroblast cell lines, monoclonal antibodies, MHC tetramers and batteries of validated PCR primers for immune-relevant genes. An important effort of the XLRRI is the development of *X. laevis* transgenic lines with fluorescent, traceable immune cells and specific immune deficiency, including the establishment of a reliable CRISPR/Cas9-mediated genome editing platform focused on immunity.

The XLRRI plays also plays a key role in understanding infectious diseases that plague amphibians worldwide and contribute to their decline. Notably, the XLRRI includes satellite facilities devoted to studying pathogenesis and immunity to ranaviruses and mycobacteria. Importantly, the XLRRI provides protocols, technical assistance and training to new as well as established investigators and students from a wide area of scientific disciplines from comparative, developmental and evolutionary immunology to field and conservation biology. Training and assistance cover general *Xenopus* breeding and husbandry, transgenesis and reverse genetics as well as *Xenopus*-specific *in vivo* and *in vitro* immunological methodologies.

## Integration and Support on Xenbase

*Xenopus* research around the world is supported by Xenbase ^4^ the *Xenopus* model organism database. Xenbase maintains, curates, and freely disseminates all of the diverse genomic, genetic, expression, and functional data for *Xenopus* and interrelates these data to human and other model organisms (Karimi et al., 2018). In addition, Xenbase allows researchers to identify *Xenopus* resources and reagents, including the transgenic and mutant lines (as detailed in this paper), as well as clones (ORFeome, plasmid, fosmid, and BAC) which are supplied by the EXRC.

Xenbase links to all the major *Xenopus* stock center websites (EXRC, NXR, XLRRI, NBRP, and CRB) from the Xenbase home page and from resource pages where appropriate. A fully searchable “Lines and Strains” module includes all of the mutant and transgenic lines and wild-type strains that are, or have been, available from NXR, EXRC and NBRP. Each *Xenopus* line has a dedicated page which summarizes targeted gene (s) or transgenic construct details, phenotype, genetic background and provenance, and provides direct links to the relevant stock centre (s) from which the line may be ordered. In the database, wild-type strains are also given “XB-LINE” identifiers so that they can be assigned as background to transgenic and mutant lines. Stable research identifiers (RRIDs) are recorded for all *Xenopus* stocks, a measure taken to promote reproducibility, rigor and transparency. Lines and strains shared between stock centers have been given unique RRIDs, as these colonies are now genetically isolated, and may be affected by genetic drift.

Importantly, Xenbase staff developed the transgenic nomenclature guidelines in consultation with the *Xenopus* stock centers and following best practice used by all other model organism databases. Using standard nomenclature is essential to ensure *Xenopus* research is accessible to the broadest possible scientific community, and improves rigor and reproducibility while also establishing provenance. These naming guidelines are posted on Xenbase ^5^. Help naming maintained lines is always available via emailing the Xenbase help desk (xenbase@ucalgary.ca) or by contacting stock center staff.

The *Xenopus* ORFeome project, funded by NICHD (R01 HD069352), was developed as a molecular toolkit to probe the cellular and genetic mechanisms underlying many human diseases using *Xenopus*. It is another resource that is fully integrated, cataloged and searchable on Xenbase ^6^. The ORFeome project produced two sets of full-length, validated, open reading frame clones, one for *X. laevis* (representing ∼ 10,250 genes, ∼7,700 with human orthologs; Grant et al., 2015), and one for *X. tropicalis* (representing ∼3,970 genes, ∼3,800 with human orthologs). Each ORFeome clone has a dedicated Xenbase page with details including gene symbol and name, full sequence, translation, availability, and links to the gene page and BLAST tool, and to the EXRC which supplies small numbers of ORFeome clones (James-Zorn et al., 2018). Similarly, all IMAGE Consortium *Xenopus* Gene Collection (XGC) clones (Morin et al., 2006) have dedicated pages with supporting sequence and other data, and these clones can be searched, filtering for only those supplied by the EXRC.

Xenbase enables efficient access to the physical resources for scientists wanting to use the *Xenopus* models; these resources are already extensive and growing rapidly as can be seen from the review above. As we write, there is still capacity in the resource centers to hold and develop new transgenic and knockout lines and we encourage all *Xenopus* users to take the opportunity to enhance their research programs by using these community facilities and taking advantage of the knowledge of their staff.

## Author Contributions

All of the authors except DG wrote and reviewed the text. DG and TI produced the data shown. MW, AA-D, SM, and CJ-Z produced the table of transgenic animals.

## Conflict of Interest Statement

The authors declare that the research was conducted in the absence of any commercial or financial relationships that could be construed as a potential conflict of interest.

## Footnotes

1 https://home.hiroshima-u.ac.jp/amphibia/xenobiores/forum/

2 https://xenopus.univ-rennes1.fr

3 https://www.urmc.rochester.edu/microbiology-immunology/xenopus-laevis.aspx

4 www.xenbase.org

5 www.xenbase.org/gene/static/tgNomenclature.jsp

6 www.xenbase.org/reagents/orf.do
